# Protocol for serious fall injury adjudication in the Strategies to Reduce Injuries and Develop Confidence in Elders (STRIDE) study

**DOI:** 10.1186/s40621-019-0190-2

**Published:** 2019-04-15

**Authors:** David A. Ganz, Albert L. Siu, Jay Magaziner, Nancy K. Latham, Thomas G. Travison, Nancy P. Lorenze, Charles Lu, Rixin Wang, Erich J. Greene, Cynthia L. Stowe, Lea N. Harvin, Katy L. B. Araujo, Jerry H. Gurwitz, Yuri Agrawal, Rosaly Correa-De-Araujo, Peter Peduzzi, Thomas M. Gill, on behalf of the STRIDE Investigators, Shalender Bhasin, Thomas M. Gill, David B. Reuben, Siobhan McMahon, Nancy K. Latham, Shehzad Basaria, Brooke Brawley, Richard Eder, Amy Larson, Lori Goehring, Molly Lukas, Scott Margolis, Thomas W. Storer, Martha B. Carnie, Priscilla Gazarian, Maureen Fagan, Peter Peduzzi, James Dziura, Denise Esserman, Erich J. Greene, Geraldine Hawthorne-Jones, Heather Allore, Margaret Doyle, Brian Funaro, Nancy Lorenze, Bridget Mignosa, Michael E. Miller, Thomas G. Travison, Peter Charpentier, Katy Araujo, Dorothy Baker, Joanne M. McGloin, Charles Lu, Haseena Rajeevan, Liliya Katsovich, Rixin Wang, Amy Shelton, Eleni Skokos, Sui Tang, Mara Abella, Carol Gordon, Teresita Pennestri, Luann Bianco, Rina Castro, Sabina Rubeck, Kenneth Rando, Barbara Foster, Karen Wu, David Nock, Crysta Collins, Leo Sherman, Stephen C. Waring, Erica Chopskie, Heather Larsen, Allise Taran, Joseph Bianco, Margaret Hoberg, Jeremy Rich, Vivian Chavez, Christine Moore, Janelle Howe, Rosario Garcia, Jocelyn Nunez, Samuel Ho, Yan Chen, Albert W. Wu, Jeremy D. Walston, Yuri Agrawal, Patti Ephraim, Tiffany Campbell, Michael Albert, Bimal Ashar, Bernard Birnbaum, Sajida Chaudry, La Toya Edwards, Scott Feeser, Naaz Hussain, Amrish Joseph, Kimberly Larsen, Alice Lee, Obafemi Okuwobi, Tara Scheck, Robert Wallace, Carri Casteel, Angela Shanahan, Julie Weldon, Anita Leveke, Charles Keller, Jeffrey Reist, Neil Alexander, Jocelyn Wiggins, Karen Burek, Tina Ledesma, Linda V. Nyquist, Nancy Gallagher, Catherine Hanson, Fred Ko, Albert L. Siu, Rosanne M. Leipzig, Christian Espino, Ravishankar Ramaswamy, Deborah West, Deborah Matza, Patricia Dykes, Hilary Stenvig, Kety FlorGomes, Taylor Christiansen, Alejandra Salazar, Laura Frain, Ariela Orkaby, Jonathan Bean, Yvette Wells, Cathy Foskett, Jerry H. Gurwitz, Allison Richards, Azraa Amroze, Lawrence Garber, Peggy Preusse, Anne McDonald, Susan L. Greenspan, Mary Anne Ferchak, Madeline Rigatti, Joseph Madia, Elena Volpi, Roxana Hirst, Eloisa Martinez, Mukaila Raji, Jay Magaziner, David A. Ganz, Pamela W. Duncan, Chad Boult, James Goodwin, Todd Manini, Kevin P. High, Lea Harvin, Cindy Stowe, Sergei Romashkan, Rosaly Correa-De-Araujo, Lyndon Joseph, Marcel Salive, Evan C. Hadley, Steven B. Clauser, David Buchner, Terry Fulmer, Susan Ellenberg, Bonita Lynn Beattie, Abby C. King, Cynthia J. Brown, Laurence Rubenstein, Mary Anne Sterling, Thomas Prohaska, Laurence Friedman

**Affiliations:** 1 Division of Geriatrics, Department of Medicine, David Geffen School of Medicine, David Geffen School of Medicine at UCLA, 11301 Wilshire Boulevard (11G), Los Angeles, CA 90073 USA; 2 Geriatric Research, Education and Clinical Center, VA Greater Los Angeles Healthcare System, Los Angeles, CA USA; 3 Icahn School of Medicine at Mount Sinai, New York, NY USA; 4 Geriatric Research, Education and Clinical Center, James J. Peters VA Medical Center, Bronx, NY USA; 5 University of Maryland School of Medicine, Baltimore, MD USA; 6 Brigham and Women’s Hospital, Boston, MA USA; 7 Marcus Institute for Aging Research, Hebrew SeniorLife, Harvard Medical School, Boston, MA USA; 8 Department of Internal Medicine, Yale School of Medicine, New Haven, CT USA; 9 Yale Center for Medical Informatics, Yale School of Medicine, New Haven, CT USA; 10 Department of Biostatistics, Yale School of Public Health, New Haven, CT USA; 11 Department of Biostatistics and Data Science, Wake Forest School of Medicine, Winston-Salem, NC USA; 12 Meyers Primary Care Institute, a joint endeavor of University of Massachusetts Medical School, Reliant Medical Group, and Fallon Health, Worcester, MA USA; 13 Department of Otolaryngology-Head and Neck Surgery, Johns Hopkins University School of Medicine, Baltimore, MD USA; 14 National Institute on Aging, Bethesda, MD USA

**Keywords:** Falls, Injuries, Adjudication

## Abstract

**Background:**

This paper describes a protocol for determining the incidence of serious fall injuries for Strategies to Reduce Injuries and Develop Confidence in Elders (STRIDE), a large, multicenter pragmatic clinical trial with limited resources for event adjudication. We describe how administrative data (from participating health systems and Medicare claims) can be used to confirm participant-reported events, with more time- and resource-intensive full-text medical record data used only on an “as-needed” basis.

**Methods:**

STRIDE is a pragmatic cluster-randomized controlled trial involving 5451 participants age ≥ 70 and at increased risk for falls, served by 86 primary care practices in 10 US health systems. The STRIDE intervention involves a nurse falls care manager who assesses a participant’s underlying risks for falls, suggests interventions using motivational interviewing, and then creates, implements and longitudinally follows up on an individualized care plan with the participant (and caregiver when appropriate), in partnership with the participant’s primary care provider. STRIDE’s primary outcome is serious fall injuries, defined as a fall resulting in: (1) medical attention billable according to Medicare guidelines with a) fracture (excluding isolated thoracic vertebral and/or lumbar vertebral fracture), b) joint dislocation, or c) cut requiring closure; OR (2) overnight hospitalization with a) head injury, b) sprain or strain, c) bruising or swelling, or d) other injury determined to be “serious” (i.e., burn, rhabdomyolysis, or internal injury). Two sources of data are required to confirm a serious fall injury. The primary data source is the participant’s self-report of a fall leading to medical attention, identified during telephone interview every 4 months, with the confirmatory source being (1) administrative data capturing encounters at the participating health systems or Medicare claims and/or (2) the full text of medical records requested only as needed.

**Discussion:**

Adjudication is ongoing, with over 1000 potentially qualifying events adjudicated to date. Administrative data can be successfully used for adjudication, as part of a hybrid approach that retrieves full-text medical records only when needed. With the continued refinement and availability of administrative data sources, future studies may be able to use administrative data completely in lieu of medical record review to maximize the quality of adjudication with finite resources.

**Trial registration:**

ClinicalTrials.gov (NCT02475850).

**Electronic supplementary material:**

The online version of this article (10.1186/s40621-019-0190-2) contains supplementary material, which is available to authorized users.

## Background

STRIDE (Strategies to Reduce Injuries and Develop Confidence in Elders) is a pragmatic cluster-randomized controlled trial that tests whether a multifactorial, individually tailored intervention delivered by a nurse fall care manager can reduce the rate of serious fall injuries when compared with usual care (Bhasin et al. 2018). Building on prior work (Schwenk et al. 2012), we defined serious fall injuries as falls resulting in: (1) medical attention billable according to Medicare guidelines with a) fracture (excluding isolated thoracic vertebral and/or lumbar vertebral fracture), b) joint dislocation, or c) cut requiring closure; OR (2) overnight hospitalization with a) head injury, b) sprain or strain, c) bruising or swelling, or d) other injury determined to be “serious” (i.e., burn, rhabdomyolysis, or internal injury). STRIDE enrolled 5451 community-dwelling participants age ≥ 70 who were at increased risk of falls and who were served by 86 primary care practices across 10 US healthcare systems (Gill et al. 2018), with follow-up continuing through March 31, 2019. Isolated thoracic and/or lumbar vertebral fractures were excluded from the primary outcome definition because the linkage between these fractures and a preceding fall could not be accurately established without a detailed medical record review, which was beyond the scope of the study.

In this report, we describe STRIDE’s protocol for central ascertainment and adjudication of potential serious fall injuries, which allows administrative data (i.e., coded data relating to the episode of care from the health system, either captured internally by the health system [i.e., encounter data], or submitted to an insurer for payment [i.e., claims data]) to be used as a first-line approach for confirming the presence and type of injuries for participants’ self-reports of falls requiring medical care. Historically, self-report has been the preferred method for assessing whether a fall occurred in community-dwelling individuals, and medical record review has been the preferred approach for verifying the presence and type of injury (Buchner et al. 1993). Thus, outcome ascertainment would typically involve obtaining medical records (i.e., copies of the notes written by healthcare professionals as documentation of a patient encounter) for confirmation of self-reported events. For STRIDE, however, obtaining medical records in all cases to verify whether falls were injurious was neither feasible financially, given a need to adjudicate 1000 to 2000 potential events, nor practical, given the vast number of locations patients could receive care. In addition, it was not financially realistic for participants to report their falls monthly, as has been advocated previously (Lamb et al. 2005). For these reasons, STRIDE investigators needed a different approach to ascertain (and then adjudicate) the primary outcome of serious fall injuries.

Administrative data can help to mitigate the limitations of both self-reported and medical record data, and have become more accessible with advances in computer technology and more detailed with the introduction of the International Classification of Diseases, Tenth Revision (ICD-10) in the United States in October 2015. Herein, we describe the STRIDE protocol for ascertaining and adjudicating potential serious fall injuries with an emphasis on describing how administrative data can be used as an alternative to a medical record review to confirm self-reported events.

## Methods

The STRIDE study’s overall design, intervention, and screening and recruitment process have been reported previously (Bhasin et al. 2018; Gill et al. 2018; Reuben et al. 2017). Ethics approval for the STRIDE protocol was provided by a specially designated central Institutional Review Board at Brigham and Women’s Hospital (Bhasin et al. 2018). The trial protocol is available at ClinicalTrials.gov (NCT02475850).

### Approach

The procedure for ascertaining a fall outcome requires that a self-report of a qualifying fall injury resulting in medical care be confirmed with either administrative data or the full text of medical records. We describe the various data sources for ascertaining information about falls in detail below.

#### Self-report

Self-report includes any information in which the participant’s (or designated surrogate’s) verbal report of an event to a study interviewer is the source of the data. In this study, self-report information stems from a telephone follow-up interview conducted every 4 months by the central STRIDE interviewer team (at Yale); interviewers are blinded to intervention/control status of participants. Interviewers first ask about falls; if one or more falls occurred, interviewers ask about any injuries related to each fall. If there are injuries, the interviewer asks about the date of injury, the injury type (and body site in the case of fractures), and use of medical care, including the name, date and type of facility or provider seen (e.g., hospital, emergency department, urgent care facility, doctor). The interviewers also ask for a verbatim description of the injury. Monthly falls calendars mailed directly to participants facilitate recollection of falls during the prior 4 months (Hannan et al. 2010).

#### Administrative data

In this study, we define administrative data sources as data routinely used for healthcare operations, either for internal purposes (e.g., tracking provider workload) by healthcare systems or for billing insurance. While providers’ billing activities may be entered into the medical record and patients’ health care encounters may be viewed in an electronic medical record, for the purposes of this study, we distinguish administrative data from medical record data in that administrative data are generally not used for direct patient care, whereas the medical record is typically accessed for provision of patient care.

In STRIDE, we distinguish encounter data and claims data as follows. Encounter data are internal data that health care systems generate for operational purposes, containing records of patients’ use of healthcare services and the reasons for the encounter (e.g., chief complaint, diagnoses). In contrast, claims are requests for payment, typically from a provider of services to an insurer. Since the vast majority of STRIDE participants were recruited after October 1, 2015, virtually all encounter and claims data use ICD-10 format for diagnoses.

The main sources of administrative data in STRIDE are the participating clinical trial sites. Most of these data are encounter data, but there are also some claims data included, typically because a clinical trial site may receive claims for payment from another entity that saw the patient on behalf of the site or because of a data-sharing agreement with other entities. The specifications for clinical trial site administrative data are available from the corresponding author upon request.

The second source of administrative data in STRIDE is claims data obtained from the Centers for Medicare and Medicaid Services (CMS) for STRIDE participants for whom we had a valid identifier to link to their data. We prospectively consented participants to link to their Medicare data at the time of enrollment and then access that data through a CMS data enclave known as the Virtual Research Data Center. A data use agreement with CMS allows extraction of specific information from the enclave (Table 1). We extract quarterly claims data from the following Medicare files: inpatient, outpatient, skilled nursing facility, hospice, home health, carrier, and durable medical equipment. In addition, we obtain the Medicare provider analysis and review (MedPAR) file, since this contains records of some Medicare Advantage hospitalizations while the inpatient file does not (Research Data Assistance Center. 2016). Nearly all data obtained from CMS pertains to STRIDE participants enrolled in traditional fee-for-service Medicare.

**Table 1:** Data extracted from Centers for Medicare and Medicaid Services enclave

Medicare enrollment information related to whether participant is enrolled in Medicare Advantage or traditional fee-for-service Medicare (including whether the participant is enrolled in Medicare Part A, Part B or both) in a given calendar month.	
Place of service information indicating the type of health care location (e.g., inpatient hospital, emergency department), including place of service codes, where applicable	
Contact information identifying the facility or professional rendering the service (e.g., facility or professional name and address, city, state, zip code and/or phone number)	
Revenue center codes and text description, where applicable	
All relevant dates, including dates of service, admission, discharge, transfer, or death, and “from” dates and “thru” dates for services spanning multiple days.	
Discharge disposition or discharge status codes, where applicable	
Diagnosis codes (ICD-9 and ICD-10)	
Diagnosis-related group codes (e.g., MS-DRG codes)	
Procedure codes (including CPT/HCPCS and ICD-9 and ICD-10 procedure codes)	
Meta-data, including the claims file and calendar year (or year and quarter, where applicable) from which the data come (e.g., carrier file from quarter 2 of 2015)	

*Abbreviations: ICD* International Classification of Diseases, *MS-DRG* Medicare Severity Diagnosis Related Groups, *CPT* Current Procedural Terminology, *HCPCS* Healthcare Common Procedure Coding System

#### Full text of medical records

Medical records are a data source that documents interactions between patients and providers, including patients’ illness histories, physical examination findings, providers’ assessment of the patient’s current diagnoses, and a plan of care. Medical records may be available in paper and/or electronic form. In some cases, medical records may overlap with administrative data in recording patients’ diagnosis codes and dates of encounters. For STRIDE, we focus on obtaining medical records data not otherwise obtainable from administrative data, including hospital history and physical notes, hospital and emergency department discharge summaries, notes from office visits, and radiographic reports.

#### Use of data sources

Self-report serves as the first source of data, since self-report is the simplest way of identifying that a fall has occurred and to obtain a date or date range for the potential injury and use of medical care. In STRIDE, self-report is the only way that a potentially qualifying event is identified. The preferred second, i.e., confirmatory, source is administrative data; these data are obtained every 6 months from study sites and quarterly from Medicare. However, medical records are an alternate second source that is used when administrative data are unavailable or when administrative data and self-report are inconsistent. Administrative data may be incomplete for a variety of reasons, including the location where the patient received care (in or outside the clinical trial site’s network), the patient’s insurance status (fee-for-service Medicare or Medicare Advantage), and the level of detail in the diagnosis and procedure codes included in the data (general code for injury versus specific code that indicates injury type). When medical records are not available from participating sites but are needed to confirm an event (typically in situations where a patient sought care from a facility outside the site’s network), we seek participants’ consent to obtain records directly from the facility where the participant reported receiving care for the fall in question.

### Adjudication process

**Fig. 1:**
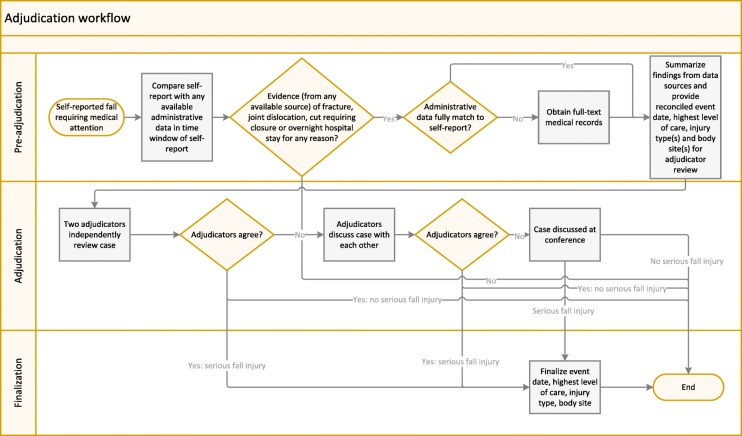
Workflow for the adjudication process

We divided the adjudication process into three parts: pre-adjudication, adjudication, and finalization. Figure 1 provides an overview of workflow; we describe each step of the process in turn below.

#### Pre-adjudication

Pre-adjudication involves obtaining, preparing, and displaying materials for adjudicators to review. The pre-adjudication process involves an adjudication coordinator (NPL, an advanced practice nurse) reviewing available self-report and administrative data, requesting records from clinical trial site coordinators when needed, and then creating a reconciled narrative of the event based on the data sources available. The adjudication coordinator reviews ambiguous events during twice-weekly phone calls with a geriatrician (DAG), who serves as overreader and verifies that events include sufficient detail to advance to adjudication. Both the adjudication coordinator and overreader are blinded to treatment assignment of participants. Custom-developed software supports the pre-adjudication process, allowing data from all sources (self-report, administrative data, and medical record data) to be viewed through a single portal.

Pre-adjudication begins with self-report data in which the participant has reported a fall resulting in medical attention. The adjudication coordinator first reviews the self-report narrative, including the circumstances of the fall and injury type, as well as any potentially matching administrative data, to determine whether the event could possibly qualify as a serious fall injury (i.e., at least one source of available information notes a potential fracture, dislocation, cut requiring closure, or overnight stay in an acute care hospital subsequent to a fall). If the event might qualify, any available administrative data are more formally matched to the participant’s self-report based on the combination of date, diagnosis codes, procedure codes, service location and/or provider/facility name, and level of care (e.g., hospitalization, emergency department visit, office visit). If there is no match, a partial match, or a discrepancy between self-report and administrative data, the adjudication coordinator requests a limited set of medical records from the coordinator at the relevant clinical trial site. Because of the pragmatic nature of this trial, if a specific medical record mapping to the index date for the self-reported event is not available, a follow-up note documenting the event in the medical record is acceptable. Confirmation from medical records is based on objective components of the record, rather than patient history, except under unusual circumstances. Objective information can come from physical exam, clinical impression, diagnoses, or a subsequent event that validates the original injury type (e.g., an encounter for suture removal validating that the injury in question required sutures be placed).

#### Adjudication

Adjudication procedures, including software capability and workflow, were refined iteratively during a pilot period with two pilot adjudicators (JHG and YA). For adjudication, physicians from each of the ten clinical trial sites are assigned a monthly batch of potentially qualifying events by the adjudication software administrator (LNH) with each event independently reviewed by two physicians (details on adjudication software and rules are provided in Additional files 1 and 2, respectively). To maintain blinding to treatment assignment, physician adjudicators do not review events from their own clinical trial site. Although additional information is collected, the two adjudicators reviewing each event are required to agree on three elements: 1) “Is there any evidence (from any data source) that an injury occurred in association with this case?” 2) “Is there any evidence (from any data source) that the injury in question occurred subsequent to a fall?” and 3) “Is this a serious fall injury?” The first two elements are screening questions to verify that an event is potentially eligible to be a primary outcome; if both adjudicators agree on a “no” answer for the first question, or a “yes” answer to the first question but a “no” answer to the second question, the event is not considered further. Events that pass screening advance to the third question, which is the key question that determines whether the event is in fact a primary outcome. To answer this question, adjudicators are asked to review whether the injury: a) resulted from a fall, b) resulted in medical attention, and c) was a qualifying injury type per the primary outcome definition.

For the third question (deciding whether the event in question is a serious fall injury), the response options are “definitely,” “highly likely,” “more likely than not (but not ‘highly likely’ or ‘definitely,’)”, “anything less than 50/50,” or “need further information.” These response options were developed during the adjudication pilot process, with the understanding that response options of “definitely” or “highly likely” would both count as a serious fall injury. Hence, a pair of responses from the two adjudicators of “definitely/definitely,” “definitely/highly likely,” and “highly likely/highly likely” counts as a match. For discrepant events in which one adjudicator indicates “definitely” or “highly likely” while the second adjudicator indicates one of the other options, the adjudication coordinator prompts them to reconcile their differences. Events that cannot be reconciled then come to a monthly case conference moderated by the geriatrician overreader in which a final decision is made after discussion with (and if needed due to ongoing disagreement, a vote by) the entire group. When a vote is needed, a simple majority determines whether the event is or is not deemed a serious fall injury.

#### Finalization

Events deemed to be serious fall injuries during adjudication proceed to finalization. The geriatrician overreader crosschecks the reconciled date of event generated during pre-adjudication, as well as the two adjudicators’ listings of the highest level of care received for the injury (overnight stay in an acute care hospital, emergency department, or other outpatient services such as an office visit), the injury type(s), and body site(s). Ambiguous events are reviewed with two other geriatricians from the central STRIDE team (ALS, TMG) until consensus is reached.

A compromise required for this pragmatic clinical trial was the need to focus on confirming one qualifying self-reported injury (typically the highest-severity injury) associated with the fall event reported by the participant, rather than confirming all injuries associated with the event. When an event has multiple confirmed injuries, we use the following hierarchy to prioritize the primary injury for that event: 1) hip fracture, 2) other fracture, 3) dislocation, 4) cut with evidence of closure, 5) hospitalization for head injury; sprain or strain; bruising or swelling; or other serious injury decided upon by adjudicators.

## Discussion

We have described an innovative adjudication protocol for a pragmatic clinical trial, involving the use of administrative data from clinical trial sites and Medicare as a first-line approach to confirm self-reports of serious fall injuries. By design, we consented participants at enrollment to permit linkage to their Medicare data. We regularly (i.e., every 3–6 months) acquire updated data from clinical trial sites and Medicare, allowing ongoing use of administrative data during the study. Other studies have explored the potential first-line use of administrative data to confirm cardiovascular outcomes (Anderson et al. 2016; Lakshminarayan et al. 2014), finding potential tradeoffs between traditional adjudication practices using medical records and use of only administrative data. We have described a hybrid approach that maximizes the benefit of administrative data while using full-text medical records when needed. We expect that interest in a hybrid approach, which may combine the best of both methods, will grow as the number of large multi-site and pragmatic trials increases, especially given relatively tight funding levels.

Adjudication for STRIDE is currently underway and is expected to conclude in January 2020. Setting up adjudication systems to accommodate multiple data sources posed challenges that had to be addressed. First, each of the 10 clinical trial sites needed to send administrative data from their health systems for review. Sites had varying degrees of difficulty accessing and providing the relevant information, as some sites were more integrated in their data collection and warehousing than others. Second, to ensure comparability of how events were confirmed via administrative data versus medical records, the procedures developed for medical record acquisition had to be refined to identify the types (and content) of materials that would be admissible as confirmatory evidence of a self-reported serious fall injury.

In the United Kingdom, the Prevention of Falls Injury Trial (PreFIT) is using administrative data to ascertain and confirm its primary outcome of peripheral fractures (excluding vertebral compression fracture) (Bruce et al. 2016). STRIDE has the added complexity of needing to adjudicate non-fracture injuries, which are more variable in how they are reported, both by participants and in administrative data. Although the Prevention of Falls Network Europe (ProFaNE) recommends not using non-fracture injuries as an endpoint (Lamb et al. 2005), STRIDE investigators chose to take a more encompassing approach primarily because of the clinical importance of many non-fracture fall-related injuries, as well as for increased statistical power.

“One-stop shopping” to obtain a complete set of administrative data from a single source would have simplified the process of data acquisition for STRIDE but is not yet feasible in the US. The available CMS data cover primarily traditional Medicare (fee-for-service) patients (Brennan, Ornstein, and Frakt 2018). Although encounter data for Medicare Advantage are now available, only 2015 data, which pre-date the vast majority of our study’s events, are currently available to researchers (Centers for Medicare and Medicaid Services. 2018). Medicare Advantage patients now represent over one-third of all Medicare enrollees (Neuman and Jacobson 2018), so having these data available will greatly facilitate future adjudication efforts. Even with full coverage of administrative data, however, we would still need to collect some medical records in full text so that discrepancies between self-report and administrative information could be resolved.

We investigated the possibility of using administrative data to uncover additional serious fall injury events not reported by participants and then confirming these events via full-text medical records. We decided not to pursue this route because of the variable coverage of administrative data, which could lead to bias in event detection due to STRIDE’s randomization of primary care practices (not participants). For example, because of ongoing data integration, one clinical trial site reported that some of its primary care practices were more likely to have encounter data available than others. This issue is minimized when self-report is used as the basis for event detection but could bias results if administrative data from the site served as the primary mechanism to identify a potentially qualifying event. Another advantage of deriving events from self-report is that the participant’s report of a fall and its consequences is currently the most accessible method for ascertaining this information (Buchner et al. 1993), which is often incompletely documented in administrative data and full-text medical records.

Limitations of this work include the lack of validation of the adjudication protocol against medical record review for all self-reported falls, regardless of whether the participant reported an injury or sought medical care for that injury. Performing such a validation was beyond the resources available in this pragmatic trial. We suspect that misclassification of events will be slightly higher than with full medical record review of all self-reported falls because some diagnoses in administrative data will be clinical and not necessarily confirmed on imaging (if applicable), but any misclassification should be at random given blinding of adjudicators to intervention/control status.

To date, over 1000 potentially qualifying events have been adjudicated. We conclude that administrative data can successfully be used for adjudication in a large multi-center clinical trial as part of a hybrid approach that pulls full-text medical records only when needed. Based on our experience, the marginal costs of using administrative data are much lower than those for obtaining the full text of medical records, but quantifying this benefit has not yet been done and is an important area for future work. With the continued refinement and availability of administrative data sources, future studies may be able to use administrative data instead of medical record review to maximize quality of adjudication in the setting of finite resources.

## Additional files

Additional file 1: STRIDE Adjudication System Instructions. (PDF 539 kb)

Additional file 2: Rules for Adjudicating. (PDF 266 kb)

Additional file 3: STRIDE Acknowledgements. (DOCX 32 kb)

## Abbreviations

CMS: Centers for Medicare and Medicaid Services
ICD-10: International Classification of Diseases, Tenth Revision
MedPAR: Medicare provider analysis and review
PreFIT: Prevention of Falls Injury Trial
ProFaNE: Prevention of Falls Network Europe
STRIDE: Strategies to Reduce Injuries and Develop Confidence in Elders

## References

[CR1] Anderson GL, Burns CJ, Larsen J, Shaw PA (2016). Use of administrative data to increase the practicality of clinical trials: insights from the women’s health initiative. Clin Trials.

[CR2] Bhasin S, Gill TM, Reuben DB, Latham NK, Gurwitz JH, Dykes P, McMahon S, Storer TW, Duncan PW, Ganz DA, Basaria S, Miller ME, Travison TG, Greene EJ, Dziura J, Esserman D, Allore H, Carnie MB, Fagan M, Hanson C, Baker D, Greenspan SL, Alexander N, Ko F, Siu AL, Volpi E, Wu AW, Rich J, Waring SC, Wallace R, Casteel C, Magaziner J, Charpentier P, Lu C, Araujo K, Rajeevan H, Margolis S, Eder R, McGloin JM, Skokos E, Wiggins J, Garber L, Clauser SB, Correa-De-Araujo R, Peduzzi P (2018). Strategies to reduce injuries and develop confidence in elders (STRIDE): a cluster-randomized pragmatic trial of a multifactorial fall injury prevention strategy: design and methods. J Gerontol A Biol Sci Med Sci.

[CR3] Brennan N, Ornstein C, Frakt AB (2018). Time to release medicare advantage claims data. JAMA.

[CR4] Bruce J, Lall R, Withers EJ, Finnegan S, Underwood M, Hulme C, Sheridan R, Skelton DA, Martin F, Lamb SE, F. I. T. S. G. Pre (2016). A cluster randomised controlled trial of advice, exercise or multifactorial assessment to prevent falls and fractures in community-dwelling older adults: protocol for the prevention of falls injury trial (PreFIT). BMJ Open.

[CR5] Buchner DM, Hornbrook MC, Kutner NG, Tinetti ME, Ory MG, Mulrow CD, Schechtman KB, Gerety MB, Fiatarone MA, Wolf SL (1993). Development of the common data base for the FICSIT trials. J Am Geriatr Soc.

[CR6] Centers for Medicare and Medicaid Services. 2018. "CMS Administrator Verma Unveils New Strategy to Fuel Data-driven Patient Care, Transparency" [Accessed 21 Nov 2018]. Available at: https://www.cms.gov/newsroom/press-releases/cms-administrator-verma-unveils-new-strategy-fuel-data-driven-patient-care-transparency.

[CR7] Gill TM, McGloin JM, Latham NK, Charpentier PA, Araujo KL, Skokos EA, Lu C, Shelton A, Bhasin S, Bianco LM, Carnie MB, Covinsky KE, Dykes P, Esserman DA, Ganz DA, Gurwitz JH, Hanson C, Nyquist LV, Reuben DB, Wallace RB, Greene EJ. Screening, recruitment, and baseline characteristics for the strategies to reduce injuries and develop confidence in elders (STRIDE) study. J Gerontol A Biol Sci Med Sci. 2018;73(11):1495–501. 10.1093/gerona/gly076 PMC6175032 30020415

[CR8] Hannan MT, Gagnon MM, Aneja J, Jones RN, Cupples LA, Lipsitz LA, Samelson EJ, Leveille SG, Kiel DP (2010). Optimizing the tracking of falls in studies of older participants: comparison of quarterly telephone recall with monthly falls calendars in the MOBILIZE Boston study. Am J Epidemiol.

[CR9] Lakshminarayan K, Larson JC, Virnig B, Fuller C, Allen NB, Limacher M, Winkelmayer WC, Safford MM, Burwen DR (2014). Comparison of Medicare claims versus physician adjudication for identifying stroke outcomes in the Women's Health Initiative. Stroke.

[CR10] Lamb SE, Jorstad-Stein EC, Hauer K, Becker C (2005). Development of a common outcome data set for fall injury prevention trials: the prevention of falls network Europe consensus. J Am Geriatr Soc.

[CR11] Neuman P, Jacobson GA (2018). Medicare advantage checkup. N Engl J Med.

[CR12] Research Data Assistance Center. 2016. "Differences between the inpatient and MedPAR files" [Accessed 21 Nov 2016]. Available at: https://www.resdac.org/articles/differences-between-inpatient-and-medpar-files.

[CR13] Reuben DB, Gazarian P, Alexander N, Araujo K, Baker D, Bean JF, Boult C, Charpentier P, Duncan P, Latham N, Leipzig RM, Quintiliani LM, Storer T, McMahon S (2017). The strategies to reduce injuries and develop confidence in elders intervention: falls risk factor assessment and management, patient engagement, and nurse co-management. J Am Geriatr Soc.

[CR14] Schwenk M, Lauenroth A, Stock C, Moreno RR, Oster P, McHugh G, Todd C, Hauer K (2012). Definitions and methods of measuring and reporting on injurious falls in randomised controlled fall prevention trials: a systematic review. BMC Med Res Methodol.

