# Supplementary material for: Protocol for serious fall injury adjudication in the Strategies to Reduce Injuries and Develop Confidence in Elders (STRIDE) study
Source: Inj Epidemiol. 2019 Apr 15;6:14. doi: 10.1186/s40621-019-0190-2 (PMC6582694; doi:10.1186/s40621-019-0190-2)

Additional File 1. STRIDE Adjudication System Instructions

The purpose of this document is to provide instructions on how to utilize the STRIDE Adjudication Management Tool for the adjudication of STRIDE outcomes.

Step 1: Login to the Management Tool via this link=> https://stride.phs.wakehealth.edu/dspLogin.cfm and enter username (your email address), and password.

The first screen you will see is the **Management Tool for Adjudicators**. This is Case List of all cases assigned to the adjudicator.

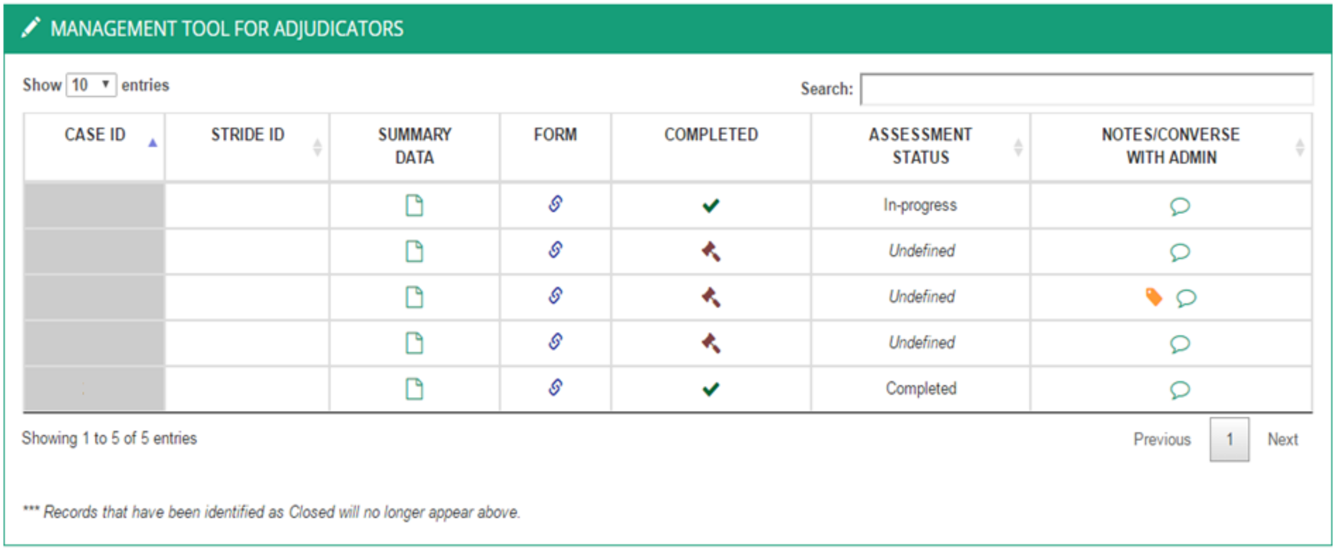

The Case List is sorted by default by the **Case ID** (column 1). The Case ID is the unique number assigned to the case. All case information is provided in the row for the Case ID. The **STRIDE ID** (column 2) is the unique number assigned to each participant. Since each participant may have more than one possible injury, there may be multiple cases for each STRIDE ID.

Summary Data (column 3) of a case is also linked in the Case List. This shows the adjudicator all the data available on each case. Click on to open the summary data. In addition, the **Form** (column 4) is the link to the adjudication form to be completed by the assigned adjudicator. Click on to open the form to complete.

When an adjudication form has any data on it the **Completed** column (column 5) will indicate whether the adjudication form has incomplete data entered on it ( ) or is blank entirely and still needs completion ( ).

After the adjudication form is completed by the adjudicator the **Assessment Status** (column 6) is reflected and entered by the adjudicator on the status from the adjudication form (the last question). This will be a good indication of which cases an adjudicator still needs to complete.

If the adjudicator needs to request help or communicate with the Administrator, under the **Notes/Converse with Admin** column (column 7) click on to post a comment to the

administrator. When the Administrator responds, the icon will change to . Mouse over the icon to view the time stamp of the last comment and whom it was made by. Click on to see notes related to the case posted by the administrator.

Other Features

In the top left of the screen the adjudicator can indicate how many cases (entries) should show on this main Case List. The adjudicator can choose 10, 25, 50 or 100 cases. In addition, in the bottom right of the screen there is a clickable link to the "previous" or "next" pages in a Case List if the adjudicator's list of cases is too long for one screen.

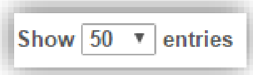

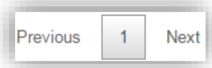

If the adjudicator wishes to sort by a different column than the default (Case ID) they can use the ▲▼ symbols in various columns where the arrows are provided. This will change the order of the cases based on the sorting clicked.

And finally, if the adjudicator would like to search or narrow down the Case List there is the search box. It will search the columns for the search parameters (Case ID, STRIDE ID, Assessment Status text) and filter out all cases that don't meet the parameters.

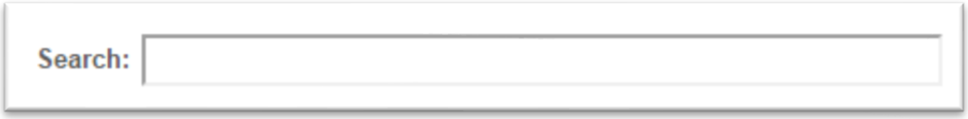

Step 2: To begin adjudication of a case the adjudicator must open and review the Summary Data for the case. Click on to open the summary data.

A new screen will open with the summary data. Depending on the browser settings the summary data may open in a new tab in the browser or a new window in the browser.

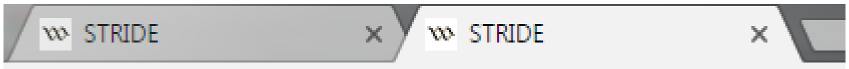

Adjudicators are advised to keep this summary data screen open on a separate screen (tab or window) in order to reference it while completing the adjudication form.

Summary Data is a summary of all the information available for the case. The Summary Data is separated into sections for each type of data source.

At the top of the Summary Data screen is what identifies the case for the adjudicator.

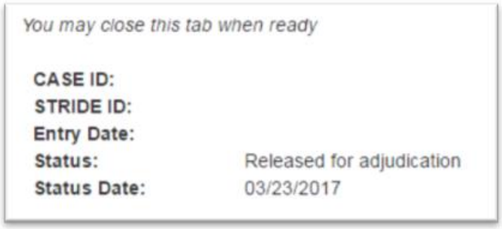

Following the Case identification section the Summary Data begins. The **Reconciled Information** is the information the STRIDE overreader has prepared for the adjudicator regarding the case. It is the overreader's best assessment of what actually happened with the case, including the date of the patient's seeking healthcare for the injury, the highest level of care received for the injury, and the injury type (with body site, if applicable).

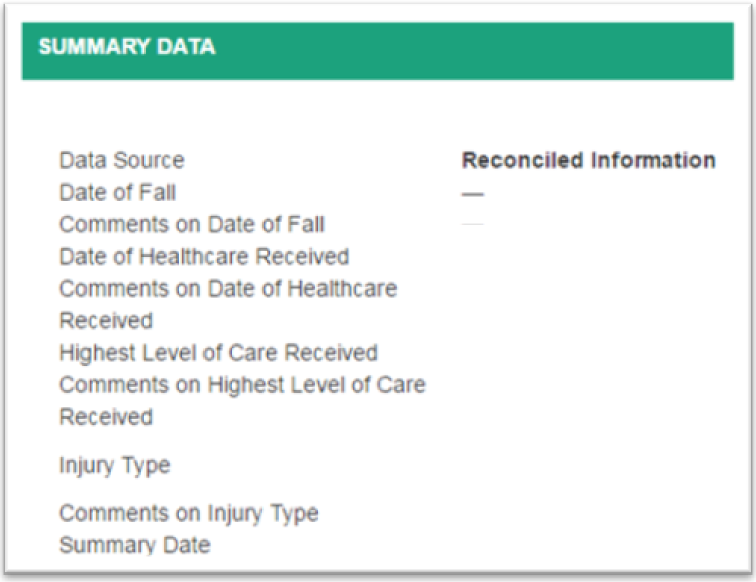

The Reconciled Information presented to the adjudicator is derived from various data sources. It can come from the patient's self-report of an injury, from encounter data submitted by clinical trial sites, from Medicare (i.e., CMS) data, and/or from the full text of medical records. With the reconciled information, the overreader will provide comments, when applicable, regarding the pertinent findings from each data source. Sometimes the various data sources will agree with each other in all aspects; other times they will not.

The job of an adjudicator is to review the summaries of the various data sources available and decide if they agree with how the case has been synthesized in the "Reconciled Information" section.

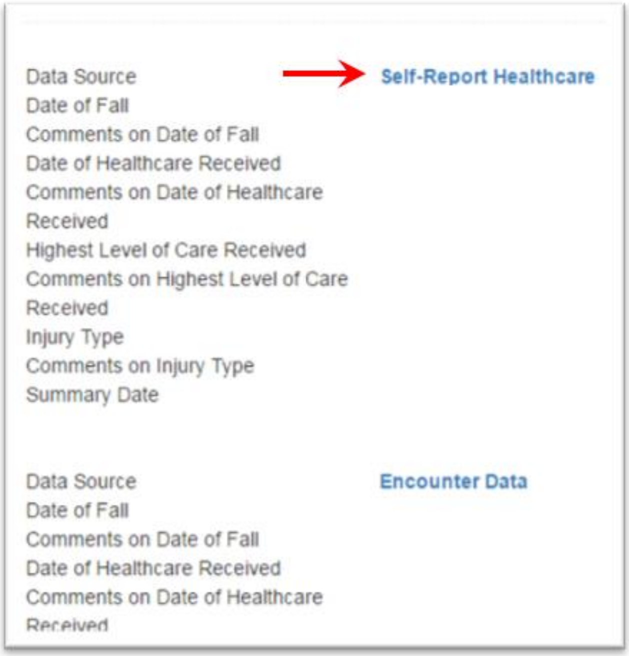

Below the reconciled information section, there will be individual summaries provided by the overreader of each data source for which data are available.

If the adjudicator chooses to further investigate the raw information from the data source, they click on the hyperlinked data source title. The hyperlinks will only be active if a given data source is available. For example, clicking on "Self-Report Healthcare" will link to and display all information pertinent to the injury as reported by the patient. Be prepared, however, to look through a large volume of data.

<u>It is not expected that the adjudicator routinely look through the raw data sources because the overreader has prepared a summary of the pertinent findings from each data source.</u>

Step 3: After the initial review of the Summary Data the adjudicator will need to open the adjudication form to complete. From the Summary Data window or tab the adjudicator should click to the primary window/tab where the Case List is located.

On the Case List, click on in the row of the desired case to open the adjudication form to complete.

To make it easier for completion of the adjudication form, adjudicators are advised to simultaneously view the Summary Data and complete the adjudication form in side-by-side windows.

After opening the adjudication form and creating a side-by-side view of the Adjudicator form and the Summary Data, adjudicators should confirm that the Case ID on the Adjudicator form matches the Case ID on the Summary Data in order to adjudicate the correct case.

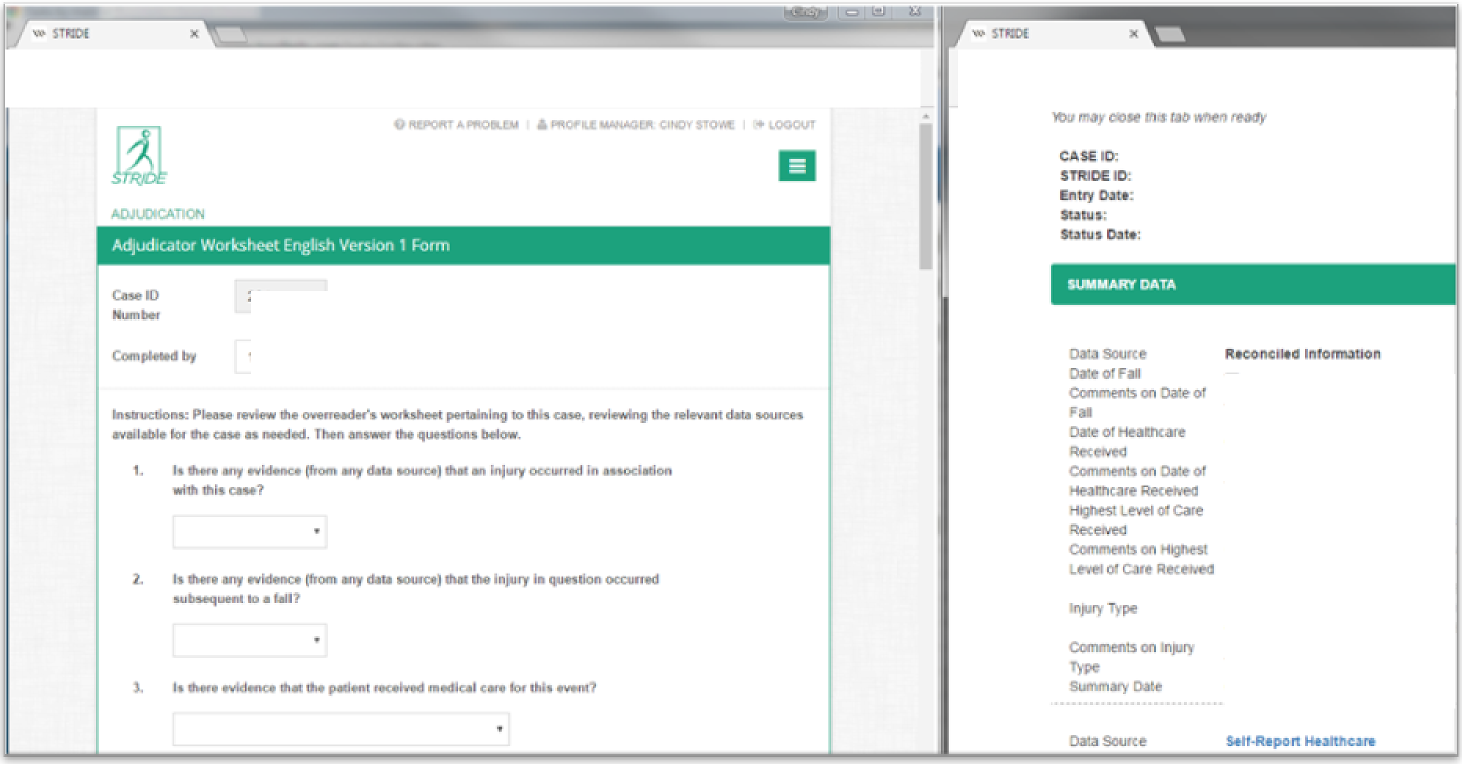

Step 4: Complete the Adjudicator form using the guidelines provided in the Rules for Adjudicating document.

Data Entry Tips

There are two methods for data entry. Adjudicator can utilize the Tab key to move to each data entry field using the number keys to select the answers (i.e., 1=Yes, 2=No) or utilize the mouse and select an answer in the dropdown.

The form is set up with rules that trigger warnings. If there are missing data (questions not answered) after saving the form at the top of the screen will be a warning. The adjudicator can go back to the missed question or “Click here to Return to List”.

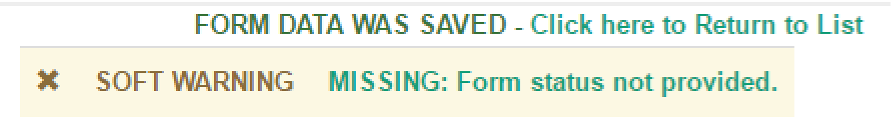

Submit the form

The last step is to click the “Submit” button to save the adjudicator’s work and submit the data.

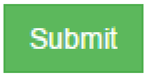

It is very important to hit the “submit” button; if the “Submit” button is not clicked, the work/data will not be saved!

After submission of the form, the form data will be saved and the screen will go back to the top of the Adjudicator form. Click on the hyperlinked “Click here to Return to List” link to go back to the Management Tool for Adjudicators and the Case List page and continue with additional adjudication or to log out.

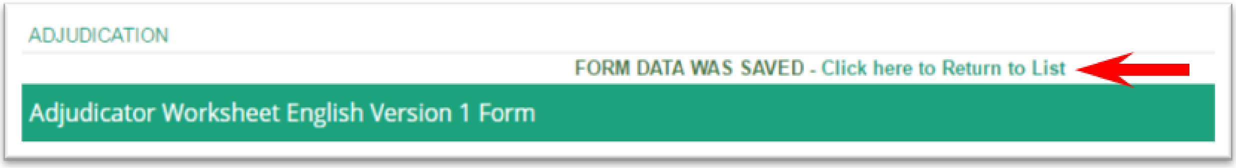

Supplement: Supplementary file 1 — STRIDE Adjudication System Instructions. (PDF 539 kb) [file 40621_2019_190_MOESM1_ESM.pdf]
