# Supplementary material for: Protocol for serious fall injury adjudication in the Strategies to Reduce Injuries and Develop Confidence in Elders (STRIDE) study
Source: Inj Epidemiol. 2019 Apr 15;6:14. doi: 10.1186/s40621-019-0190-2 (PMC6582694; doi:10.1186/s40621-019-0190-2)
Supplement: Supplementary file 2 — Rules for Adjudicating. (PDF 266 kb) [file 40621_2019_190_MOESM2_ESM.pdf]

Additional file 2: Rules for Adjudicating
Last update: 03/22/18

Question 1: "Is there any evidence (from any data source, including self-report) that an injury occurred in association with this case?"

This question verifies that at least a minimal amount of evidence supports a possible injury. Err on the side of saying "yes" if you're not sure.

- Note that syncope or presyncope are not injuries in and of themselves. They are potential mechanisms of injury.
- Also, a patient report of pain with concern for a possible injury is not an injury.

Note that you and the other adjudicator are required to agree on question 1. Lack of agreement will trigger the administrator to ask you and the other adjudicator to discuss the case and revote on questions 1, 2 and 6. When revoting on question 1, use the "FINAL-yes" or "FINAL-no" options to indicate your final response. If you and the other adjudicator still do not agree after discussion, then the case will either be reviewed by a third adjudicator or be sent directly to the overall adjudication committee for review, to make a final decision.

Question 2: "Is there any evidence (from any data source, including self-report) that the injury in question occurred subsequent to a fall?"

This question verifies that at least a minimal amount of evidence links the injury to a fall. Again, err on the side of saying "yes" if you're not sure.

- Remember that the definition of a fall is broad: "defined as an unexpected event in which the participant comes to rest on the ground, floor, or lower level." The mechanism of the fall (e.g., mechanical versus from loss of consciousness or other intrinsic cause) does not affect whether the event is considered a fall.
- Note that the definition above implies the fall must precede the injury, and not the other way around.
- Vehicle collisions with subsequent trauma are considered mutually exclusive mechanisms of injury from falls, and so question 2 should be answered "no" for vehicle collisions.

Note that you and the other adjudicator are required to agree on question 2. Lack of agreement will trigger the administrator to ask you and the other adjudicator to discuss the case and revote on questions 1, 2 and 6. When revoting on question 2, use the "FINAL-yes" or "FINAL-no" options to indicate your final response. If you and the other adjudicator still do not agree after discussion, then the case will either be reviewed by a third adjudicator or be sent directly to the overall adjudication committee for review, to make a final decision.

Question 3: "Is there evidence that the patient received medical care for this event?"

This question is focused on determining whether a licensed independent provider who can bill Medicare (e.g., physician, nurse practitioner, physician assistant) provided care directed toward the event in question.[1]

Answer "yes" to this question if the following two criteria are met. Answer "no" if at least one of the following two criteria is not met. If you are uncertain, choose the "uncertain/not enough information to decide" option and describe the reason for your uncertainty.

---

[1] Generally speaking, healthcare encounters that fall within a month on either side of the patient's reported date of seeking healthcare for the event are treated as being a potential match for the patient's reported event. ICD-10 codes may say "initial encounter" even for encounters for subsequent treatment, so the designation of "initial encounter" versus "subsequent encounter" need not factor in to a decision about a potential match. In addition, note that there may be a latency period between the initial fall and seeking medical attention.

1. The medical care needs to fall under a list of services that are covered by Medicare. From a practical standpoint, if encounter data, claims data or medical record data are available that match the injury for your case, you can treat such data as evidence of a Medicare-covered service. If you need to do a deeper dive, the Centers for Medicare and Medicaid Services (CMS) have a website that shows what Medicare does and doesn't cover*:* https://www.medicare.gov/what-medicare-covers/.
    - Services not covered by Medicare:
        - Acupuncture
        - Outpatient dental care
    - Services covered by Medicare under limited circumstances:
        - Chiropractic care, for correction of "manual manipulation of the spine if medically necessary to correct a subluxation"
2. There needs to be evidence that the service did result or could have resulted in a separate encounter billable to Medicare.[2] Again, if encounter data, claims data or medical record data match the injury for your case, you can treat that as evidence of a billable encounter.[3]
    - Services that would not count:
        - Services rendered at home by non-billing providers as part of ongoing care that was arranged prior to the injury (e.g., hospice or home health nurse visit)
        - Services rendered at home by on-site non-billing providers (e.g., nurse on site at assisted living facility).
        - Telephone calls (audio only, no video)[4]

Question 4: "What is the highest level of medical care received for this event?"

We recommend that generally speaking, you answer this question based on the reconciled information provided by the overreader. When data sources conflict about the level of care, the overreader will reconcile the data using the highest-quality objective data source available (encounter data, claims data, or medical records). Unless you see a reason to disagree, you can use the reconciled information.

- Use "overnight stay in acute care hospital (including inpatient and observation)" for any cases with a hospital admission.
- Use "emergency department visit or same-day (i.e., not overnight) hospital observation stay" when the highest level of care noted by the overreader is an emergency department visit. The overreader distinguishes urgent care and emergency department visits based on the billing entity listed in an objective source of data (encounter data, claims data, or medical records).
- Use "other outpatient services (e.g., skilled nursing facility, urgent care, office visit)" for urgent care visits, office visits, and physical/occupational therapy visits.
- Use "other inpatient services (describe)" for admissions to acute rehabilitation facilities, long-term acute care hospitals, and critical access hospitals and provide a description.

Question 5: "What type(s) of injuries occurred with this event? Specify all that apply.

Regarding injury types: The overreader follows specific rules in indicating the reconciled injury types for each case. An injury type is only listed by the overreader in the reconciled information if it is confirmed by an objective source of data. Note that you should only list confirmed injury types in Question 5. Patient-reported injury types

---

[2] The following providers can bill Medicare independently: Anesthesiology Assistants; Audiologists; Certified Nurse-Midwives; Certified Registered Nurse Anesthetists; Clinical Nurse Specialists; Clinical Psychologists; Clinical Social Workers; Mass Immunization Roster Billers, individuals; Nurse Practitioners; Physical/Occupational Therapists in private practice; Physicians (Doctors of Medicine or Osteopathy, Doctors of Dental Medicine; Dental Surgery; Podiatric Medicine; or Optometry); Physician Assistants; Psychologists practicing independently; Registered Dietitians or Nutrition Professionals; Speech-Language Pathologists

[3] This includes encounters for imaging studies that occur independent of face-to-face visits with the ordering provider.

[4] Video visits could potentially count if there is evidence of physical examination via video (e.g., identification of ecchymosis), but these cases should be discussed in conference.

that are not confirmed should not be listed, except in rare instances where the self-report data are more detailed and credible than other sources. For confirmed injury types, list all that apply.

Choosing injury types:

- Choose “fracture” for each confirmed fracture
- Choose “joint dislocation” for each confirmed dislocation or subluxation unless there is a combined fracture/dislocation injury (in which case “fracture” should be selected).
- Choose “cut with closure” if glue, staples, or sutures were required to close the cut/laceration, or if a procedure code is present in encounter or claims data indicating closure. (Wound dressings, steri-strips, and bandages do not count as closure.)
- Choose “cut without closure” if there is a cut/laceration but there is no definitive evidence of a medical procedure to close the cut. Abrasions should be classified in this category.
- Choose “head injury” for injuries (e.g., contusions, concussions, intracranial bleeding subsequent to head trauma) that cannot be more specifically described using the “fracture” or the “cut with closure” category.
  - Examples of injuries that should not be classified under “head injury”: Facial fractures and skull fractures should be categorized under the “fracture” category with appropriate body site specified. Lacerations of the scalp or face requiring closure should be categorized under the “cut with closure” category with appropriate body site specified.
  - Examples of injuries that should be classified under “head injury”: bruising and swelling of the head (e.g., scalp hematoma or contusion) would be classified under “head injury” rather than “bruising and swelling.”
- Choose “sprain or strain” for appropriate muscle, ligament, or tendon injuries not otherwise involving a fracture or dislocation.
- Choose “bruising or swelling” for bruises, hematomas, contusions or injury-induced swelling in non-head locations.
- Choose “other” to describe another injury, such as a burn.

Regarding body sites: Not all injury types may have a clear body site, but most will. The overreader follows specific rules in indicating the reconciled body site for each injury type. Body sites are based on an objective source of data (encounter data, claims data, or medical record data) wherever possible. Note that all fractures must have a specific body site listed, and if there are fractures at multiple body sites, each fracture/body site combination should be listed as a separate injury in the drop-down menu.

Choosing a body site:

Body site locations include:

- head/skull – includes all locations on the head except facial locations
- face – includes anywhere on the face, including nose
- neck – includes cervical spine
- collar bone – use for clavicle
- ribs – use for ribs
- tailbone – use for coccyx
- back/spine vertebrae (non-neck and non-tailbone) – use for lumbar vertebral and thoracic vertebral locations
- shoulder/upper arm – includes proximal humeral or mid-humeral locations (upper end of humerus, surgical neck of humerus, greater/lesser tuberosities of humerus, shaft of humerus).
- shoulder blade – use for scapula, including acromial and coracoid processes
- elbow – includes lower humeral locations (lower end of humerus, supracondylar, intercondylar, condyles, epicondyles, transcondylar), upper end of ulna (including olecranon, coronoid process), upper end of radius (radial head, radial neck)

- lower arm – includes shaft of ulna, shaft of radius, unspecified forearm locations
- wrist – includes lower end of radius (distal radius), distal ulna (lower end of ulna, ulnar styloid), carpal bones
- hand/fingers – includes metacarpals and phalanges of hand
- pelvis -- includes ilium, acetabulum, pubis, ischium
- hip – includes proximal femur, including femoral head, femoral neck, intertrochanteric and subtrochanteric locations
- upper leg/femur – includes femoral shaft
- knee – includes distal femur (supracondylar and condylar locations, lower epiphysis), patella, and proximal tibia (tibial spine, tibial plateau, tibial condyles, tibial tuberosity)
- lower leg – includes tibial shaft, upper end of fibula, shaft of fibula
- foot/toes – includes calcaneus, tarsal bones, metatarsals, and phalanges of feet
- ankle – includes distal tibia (malleoli), distal fibula (malleoli), talus
- other – use for other body sites not corresponding to the ones above, and add text description

Question 6: "Is this a serious fall injury?"

A serious fall injury requires that three elements be present:

1. The injury resulted from a fall (defined as an unexpected event in which the participant comes to rest on the ground, floor, or lower level) – <u>for this element, any single source of data, including self-report, is sufficient, as long as the fall as a mechanism of injury is not contradicted by another source of data suggesting a different mechanism. We only require a single source of data because falls are often not reported in objective sources of data such as encounter data or medical records.</u>
2. The injury resulted in medical attention – <u>defined as noted in question 3 above</u>.
3. The injury was one of these types: fracture [excluding thoracic vertebral and/or lumbar vertebral fractures of any type], joint dislocation, cut requiring closure, head injury, sprain or strain, bruising or swelling, or other major sequelae (e.g., burns, rhabdomyolysis, internal injuries, hypothermia)[5] – <u>your classification of the injury type in question 5 should help you answer this question.</u>[6]

The choices for answering Question 6 are:

a. Definitely (<u>no uncertainty</u>)
b. Highly likely (<u>a little uncertainty but very close to "definitely" – no major uncertainties</u>)
c. More likely than not (but not "highly likely" or "definitely") – <u>on balance this is more likely to be a serious fall injury than not, but there is some significant uncertainty that throws the decision into question</u>
d. Anything less than fifty-fifty – <u>this is probably or definitely not a serious fall injury</u>
e. Need further information

The two adjudicators are required to agree on question 6.

- Positive agreement means: any combination of a. and b. above (a/a, a/b or b/b)
- Negative agreement means: any combination of c. and d. above (c/c, c/d or d/d)

---

[5] Note that the "other major sequelae" category is not limited to the examples shown. If in doubt about whether a particular injury should count towards this category, please request that the case go to central adjudication by committee (see question 10).

[6] As noted in the guidance for Question 5, injury types only count if there is positive confirmation by an objective source of data. This includes claims/encounter data OR selected parts of a full-text medical record (physical exam, diagnoses, assessment/impression, plan, procedures performed). The "OR" statement is very important: it means that if both claims/encounter data and full-text medical record data are available, positive confirmation from either source (regardless of the other source) can be considered confirmation of the patient's report. If claims/encounter data confirm an injury type but full-text medical records disagree, the claims/encounter data prevail.

Lack of agreement will trigger the administrator to ask the adjudicators to reconcile the case and revote on questions 1, 2 and 6. When revoting on question 6, use the options in the drop-down menu labeled as "FINAL" to indicate your final response. If you and the other adjudicator still do not agree, then the case will either be reviewed by a third adjudicator or be sent directly to the overall adjudication committee for review, to make a final decision.

Question 7: "Was there is evidence that the injury event was a nosocomial injury? (e.g., occurring while the patient was in a hospital or nursing home setting)"

Any evidence that the injury occurred in the context of a healthcare setting should count here. Provide a detailed explanation in the text box of the circumstances of the injury. This information may be used in sensitivity analyses to exclude events where the patient's involvement in the fall is unclear (e.g., a fall occurring while a patient was being assisted by staff).

Question 8: "Were you unblinded to intervention or control group status by reviewing this case?"

If answering "yes," please explain how the unblinding occurred, which data source was the source of the unblinding, and, if the unblinding occurred in a medical record document, where in the medical records unblinding information was seen.

- All adjudicators should remain blinded to which group a patient is in. If you are unblinded, the administrator will need to know how this happened and may need to reassign the case after the case is properly blinded.

Question 9: "Do you have any additional comments regarding the injury or the adjudication of this injury?"

Please let us know any special circumstances of this case that could not properly be explained in one of the more specific text boxes earlier.

Question 10: "Do you feel this case needs to go to central adjudication by committee?"

By selecting "yes" you are indicating that the case needs a full committee review. Check this option if you and your fellow adjudicator could not come to consensus on some aspect of the case, or if you think there is some aspect of the case that would be worth discussing in a larger group because of its complexity.
