# Supplementary material for: Protocol for serious fall injury adjudication in the Strategies to Reduce Injuries and Develop Confidence in Elders (STRIDE) study
Source: Inj Epidemiol. 2019 Apr 15;6:14. doi: 10.1186/s40621-019-0190-2 (PMC6582694; doi:10.1186/s40621-019-0190-2)
Supplement: Supplementary file 3 — STRIDE Acknowledgements. (DOCX 32 kb) [file 40621_2019_190_MOESM3_ESM.docx]

**Additional File 3:** STRIDE Acknowledgements

**The STRIDE Study Team**

*Joint Principal Investigators:*

Shalender Bhasin MB, BS, Communicating PI and Chair of the Steering and Publications Committees, and Central Project Management (Brigham and Women’s Hospital, Harvard Medical School, Boston, MA)

Thomas M. Gill MD, PI and Chair of Protocol and Recruitment Committees (Yale School of Medicine, New Haven, CT)

David B. Reuben MD, PI and Chair of Intervention Committee (David Geffen School of Medicine at UCLA, Los Angeles, CA)

*School of Nursing, University of Minnesota*

Siobhan McMahon PhD MPH GNP-BC (Nursing Director)

*Central Project Management, Brigham and Women’s Hospital, Boston, MA:*

Shalender Bhasin (PI), Nancy K. Latham PhD PT (Study Director), Shehzad Basaria MD (Chief Medical Safety Officer), Brooke Brawley (cIRB Liaison), Richard Eder BA, Amy Larson MHA (Administrative Director), Lori Goehring BA (Study Manager), Molly Lukas BS, Scott Margolis MBA, Thomas W. Storer PhD, Martha B. Carnie AS, Priscilla Gazarian PhD RN [University of Massachusetts Boston, Boston, MA], Maureen Fagan DNP MHA FNP-BC [University of Miami Health System, Miami, FL]

*Data Coordinating Center, Yale University, New Haven, CT:*

Peter Peduzzi (Director) PhD, James Dziura PhD, Denise Esserman PhD, Erich J. Greene PhD, Geraldine Hawthorne-Jones, Heather Allore PhD, Margaret Doyle MPH, Brian Funaro, Nancy Lorenze DNSc, Bridget Mignosa, Michael E. Miller PhD (Wake Forest), Thomas G. Travison PhD (Harvard), Peter Charpentier MPH (Chair of Data Management Committee), Katy Araujo MPH (Co-Chair of Data Management Committee)

*Yale School of Medicine, New Haven, CT:*

*Geriatric Medicine:*

Dorothy Baker PhD

*Recruitment and Assessment Center:*

Joanne M. McGloin MDiv, MS, MBA, Charles Lu PhD, Haseena Rajeevan PhD, Liliya Katsovich MA MBA CCRP, Rixin Wang PhD, Amy Shelton MPH, Eleni Skokos BS MS, Sui Tang BS, Mara Abella, Carol Gordon, Teresita Pennestri, Luann Bianco, Rina Castro, Sabina Rubeck, Kenneth Rando, Barbara Foster, Karen Wu, David Nock, Crysta Collins, Eloisa Martinez (UTMB), Leo Sherman (Mt. Sinai)

*Clinical Trial Sites*

*Essentia Health*, Duluth, MN

Site Principal Investigator: Stephen C. Waring, DVM, PhD, Fall Care Managers: Erica Chopskie BS, RN and Heather Larsen BA, RN, Allise Taran MPH, Joseph Bianco MD, Margaret Hoberg CNP, Hillary Henzler

*HealthCare Partners*, El Segundo, CA

Site Principal Investigator: Jeremy Rich, DPM, Fall Care Manger: Vivian Chavez RN, Christine Moore, Janelle Howe, Rosario Garcia, Jocelyn Nunez, Samuel Ho MD, Yan Chen, MD

*Johns Hopkins Medicine*, Baltimore MD

Site Principal Investigator: Albert W. Wu MD MPH, Jeremy D. Walston, MD, Yuri Agrawal MD, Patti Ephraim MPH; Fall Care Manager: Tiffany Campbell BSN, RN, BMTCN, OCN; Johns Hopkins Community Physicians: Michael Albert MD, Bimal Ashar MD, Bernard Birnbaum MD, Sajida Chaudry MD, LaToya Edwards MD, Scott Feeser MD, Naaz Hussain MD, Amrish Joseph MD, Kimberly Larsen MD, Alice Lee MD, Obafemi Okuwobi MD, and Tara Scheck MD.

*Mercy Health Network* (Des Moines, IA*) and* *University of Iowa* (Iowa City, IA)

Site Principal Investigator: Robert Wallace MD, MSc, Co-Site Principal Investigator: Carri Casteel PhD, Fall Care Manager: Angela Shanahan RN-BC, BSN, Julie Weldon MSN RN, Anita Leveke RN, BSN, CEN, Charles Keller MD, Jeffrey Reist PharmD

[*Michigan Medicine*, University of Michigan](http://www.ufl.edu/), Ann Arbor, MI

Site Principal Investigator: Neil Alexander MD, Jocelyn Wiggins BM BCh, Fall Care Managers: Karen Burek RN MS ANP-BC and Tina Ledesma RN BSN, Linda V Nyquist PhD, Nancy (Amby) Gallagher, PhD, APRN-BC, Catherine Hanson BA

[*Mount Sinai Health System*](http://www.northwestern.edu/), New York, NY

Site Principal Investigator: Fred Ko MD MS, Albert L. Siu MD MPH, Rosanne M. Leipzig MD PhD, Christian Espino BA, Ravishankar Ramaswamy MD MS, Fall Care Managers: Deborah West RN BS and Deborah Matza RN MPH

[*Partners Healthcare*](http://www.uab.edu/), Boston, MA

Site Principal Investigator: Patricia Dykes, RN, PhD, MA, Hilary Stenvig BS, Kety FlorGomes BA, RN BSN , Taylor Christiansen BS, Alejandra Salazar PharmD, Laura Frain MD, Ariela Orkaby MD MPH, Jonathan Bean MD Fall Care Managers: Yvette Wells RN and Cathy Foskett RN

*Reliant Medical Group*, Worcester, MA

Site Principal Investigator: Jerry H. Gurwitz MD, Allison Richards BA, Azraa Amroze BS, Lawrence Garber MD, Fall Care Managers: Peggy Preusse RN and Anne McDonald RN

*University of Pittsburgh Medical Center*, Pittsburgh, PA

Site Principal Investigator: Susan L. Greenspan, MD, Fall Care Manager: Mary Anne Ferchak RN BS, Madeline Rigatti, Joseph Madia

*University of Texas Medical Branch* at Galveston, Galveston, TX

## Site Principal Investigator: Elena Volpi MD, Fall Care Manager: Summer Chapman RN MSN, Roxana Hirst MS CCRP, Eloisa Martinez BS, CCRP, Mukaila Raji MD, MS

*STRIDE Committee Chairs:*

***Clinical Trial Sites****:* Jerry H. Gurwitz MD*;*

***Outcomes****:* Jay Magaziner MSHyg PhD (University of Maryland) and Albert L. Siu MD MPH*;*

***Adjudication****:* David A. Ganz MD PhD (UCLA and VA Greater Los Angeles Healthcare System)

***Steering:*** Shalender Bhasin MB, BS

***Protocol:*** Thomas M. Gill MD

***Screening; Recruitment and Retention***: Joanne M. McGloin M Div, MS, MBA and Thomas M. Gill MD

***Data Management and IT***: Peter Charpentier MPH, Katy Araujo MPH

***Intervention***: David B. Reuben MD

***Safety:*** Shehzad Basaria MD

***Falls Care Managers***: Siobhan McMahon PhD RN (University of Minnesota);

***National Patient and Stakeholder***: Maureen Fagan DNP MHA FNP-BC (Chair), Martha B. Carnie AS (Co-Chair), Catherine Hanson BA

***Physical Components***: Pamela W. Duncan PhD, PT (Wake Forest University) and Thomas W. Storer PhD

***FCM Training***: Chad Boult MD MPH MBA (Johns Hopkins) and Priscilla Gazarian PhD RN

***Ancillary Studies***: James Goodwin MD (UTMB) and Todd Manini PhD (University of Florida)

***Publications***: Shalender Bhasin MB, BS and Peter Peduzzi PhD

*Wake Forest University, Winston-Salem NC*

Kevin P. High MD MS, Lea Harvin, Cindy Stowe

*National Institute on Aging, Bethesda, Maryland:*

Program Officer: Sergei Romashkan MD, Scientific Officer: Rosaly Correa-De-Araujo MD MS PhD, Lyndon Joseph PhD, Marcel Salive MD MPH, Evan C. Hadley MD

*Patient Centered Outcomes Research Institute (PCORI), Washington, D.C.*

Steven B. Clauser PhD MPA

**Data and Safety Monitoring Board**

A 9-member Data and Safety Monitoring Board, appointed by the National institute on Aging, oversaw study’s progress and safety, and included:

David Buchner MD MPH(Chair); Terry Fulmer PhD RN FAAN; Susan Ellenberg PhD; Bonita Lynn Beattie MPT MHA; Abby C. King PhD; Cynthia J. Brown MD MSPH; Laurence Rubenstein MD; Mary Anne Sterling CEA; Thomas Prohaska PhD; [Laurence](mailto:l.m.friedman@verizon.net) Friedman MD

## Funding Support

The STRIDE study was funded primarily by the Patient Centered Outcomes Research Institute (PCORI), with additional support from the National Institute on Aging (NIA) at NIH. Funding is provided and the award managed through a cooperative agreement (5U01AG048270) between the NIA and the Brigham and Women’s Hospital. The project is part of the Partnership for Fall Injuries Prevention between the NIA and PCORI. This research is partially supported by the Boston Claude D. Pepper Older Americans Independence Center at Brigham and Women’s Hospital (P30-AG013679) and Harvard Catalyst | The Harvard Clinical and Translational Science Center (National Center for Research Resources and the National Center for Advancing Translational Sciences, National Institutes of Health Award UL1 TR001102) and financial contributions from Harvard University and its affiliated academic healthcare centers. Support was also provided by the Claude D. Pepper Older Americans Independence Centers at UCLA (P30AG028748); Yale (P30AG021342); Mt Sinai (P30AG2874106); UTMB (P30AG024832), University of Michigan (P30AG024824) and Wake Forest (P30AG021332). Mt Sinai also received support through a grant from the New York Academy of Medicine. Additional support at Yale University was provided by the NIH/National Center for Advancing Translational Sciences Clinical and Translational Science Awards program (UL1TR000142) and an Academic Leadership Award (K07AG043587) to Dr. Gill from the National Institute on Aging. Dr. McMahon was supported by grants KL2TR000113 and UL1TR000114. The University of Michigan also received support from Michigan Medicine, its academic healthcare system. The content of this publication is solely the responsibility of the authors and does not necessarily represent the official views of the National Institutes of Health.
